# NR4A1 is associated with chronic low-grade inflammation in patients with type 2 diabetes

**DOI:** 10.3892/etm.2014.1958

**Published:** 2014-09-11

**Authors:** QI HUANG, JUNLI XUE, RUNMEI ZOU, LI CAI, JING CHEN, LI SUN, ZHE DAI, FAN YANG, YANCHENG XU

**Affiliations:** Department of Endocrinology, Zhongnan Hospital of Wuhan University, Wuhan, Hubei 430071, P.R. China

**Keywords:** diabetes, inflammation, nuclear receptor subfamily 4 group A member 1, nuclear factor-κB, IκBα

## Abstract

Type 2 diabetes (T2D) is a common disorder characterized by chronic low-grade inflammation. In the present study, the expression levels of nuclear receptor subfamily 4 group A member 1 (NR4A1) and the correlation with inflammatory cytokine production and free fatty acids (FFAs) in patients with T2D and healthy participants were investigated. NR4A1 expression levels in peripheral blood mononuclear cells (PBMCs) from patients with T2D (n=30) and healthy controls (n=34) were analyzed. In addition, the levels of fasting blood glucose (FBG), fasting plasma insulin (FIN), FFAs, total cholesterol (TC), triglyceride (TG), high-density lipoprotein-cholesterol (HDL-C) and low-density lipoprotein-cholesterol (LDL-C) were analyzed, and the homeostasis model assessment (HOMA) was used to estimate the insulin resistance (IR). Additionally, PBMCs from healthy subjects were cultured with or without 250 μM palmitic acid (PA). Levels of NR4A1, tumor necrosis factor-α (TNF-α) and interleukin-6 (IL-6) in the PBMCs were also analyzed. The basal expression levels of NR4A1, TNF-α and IL-6 were higher in the T2D patients when compared with the controls. In addition, the levels of FFAs, TG and LDL-C, as well as the HOMA-IR, were higher in T2D patients. Furthermore, NR4A1 expression was demonstrated to positively correlate with the HOMA-IR and the levels of FFAs, TNF-α, IL-6, FIN and FBG. Furthermore, 250 μM PA stimulation was shown to increase NR4A1 expression and the secretion of inflammatory cytokines in the cultured PBMCs. Therefore, increased NR4A1 expression levels are correlated with a chronic low-grade inflammatory state and the disorder of lipid metabolism in patients with T2D.

## Introduction

The incidence of diabetes is increasing, with the disease affecting ~347 million adults worldwide (1), which is projected to increase to 552 million by 2030 (2). Type 2 diabetes (T2D) is the most common type of diabetes. Increasing evidence indicates that inflammation is involved in the pathogenesis of T2D, with levels of C-reactive protein (CRP), a biomarker of inflammation, increased in patients that are obese and diabetic (3–5). Levels of proinflammatory cytokines, including interleukin (IL)-1β, IL-6, tumor necrosis factor-α (TNF-α) and plasminogen activator inhibitor (PAI-1), are also increased in patients who are obese and diabetic (3–7). Prospective studies have demonstrated that higher plasma levels of CRP, fibrinogen, IL-6 and PAI may be used to predict the risk of developing T2D (3,6–9). In addition, alterations in the leukocyte count are involved in T2D. Investigations by Nakanishi *et al* (10) indicated that a higher white blood cell count may predict the development of impaired fasting glucose and T2D. Additionally, an impaired T-cell balance has been observed in patients with T2D, characterized by CD4^+^CD28 null T-cell expansion and CD4^+^CD25^+^Foxp3^+^ regulatory T-cell reduction (11). Furthermore, results from clinical trials have shown that the administration of anti-inflammatory agents, such as IL-1 antagonists, in patients with T2D significantly lowered blood glucose levels, as well as CRP, IL-6 and other inflammatory biomarkers (12,13).

TNF-α and IL-6 have been shown to impair insulin signaling pathways (14), blunting the response of the liver, adipose tissue and skeletal muscle to insulin. Increasing evidence has demonstrated that inflammation is also involved in islet β-cell dysfunction (15). Therefore, the hypothesis that T2D is a chronic low-grade inflammatory disease has arisen (4,5).

Nuclear receptor subfamily 4 group A member 1 (NR4A1), also known as Nur77, nerve growth factor I-B (NGFI-B) and TR3, is encoded by the *Nr4a1* gene and is a member of the NR4A nuclear receptor superfamily (16,17). The receptor also belongs to the orphan nuclear family (16,17). The domain structure of NR4A1 is similar to other nuclear receptors, containing an N-terminal activating function-1 domain, a zinc finger DNA-binding domain and a C-terminal ligand-binding domain (16,17). NR4A1 is reported to have multiple biological functions, regulating cell proliferation, differentiation, apoptosis, development, metabolism and immunity (16,18–20). The receptor exerts these physiological functions through expression regulation, post-translational modification and subcellular localization (21).

Previous studies have indicated that NR4A1 exerts effects on inflammatory processes (17,22–26). Macrophages stimulated by oxidized low-density lipoprotein (oxLDL), lipopolysaccharide (LPS) and TNF-α result in a higher transcription of *Nr4a1* (23,24,26). Overexpression of *Nr4a1* in RAW macrophages induces several inflammatory cytokines (23), including IκB kinase (IKK)i/IKKɛ. Furthermore, previous studies have indicated that NR4A1 is expressed by macrophages in human atherosclerotic lesions (24,25). You *et al* (22) demonstrated that NR4A1 suppressed proinflammatory activation in endothelial cells (ECs).

However, the association between NR4A1 expression and the chronic low-grade inflammatory state in patients with T2D remains unknown. Therefore, the aim of the present study was to investigate the expression levels of NR4A1 in human peripheral blood mononuclear cells (PBMCs), which are partly derived from the circulatory system and can be regarded as an insight into inflammation. In addition, the association between NR4A1 levels and inflammation-related parameters was analyzed, as well as the alteration in NR4A1 expression in palmitic acid (PA)-treated PBMCs.

## Materials and methods

### Patients with T2D and healthy subjects

The study was performed in accordance with and with approval from the Ethics Committee of Zhongnan Hospital of Wuhan University (approval no. 2012012; Wuhan, China). According to medical history and clinical examination, 64 participants, including 34 healthy subjects and 30 patients with newly diagnosed T2D, were recruited from the Zhongnan Hospital of Wuhan University. Written informed consent was obtained from the patient. All the participants underwent a complete physical examination and laboratory tests.

Exclusion criteria were as follows: i) Aged <20 or >65 years; ii) body mass index (BMI) of <15 or >32 kg/m^2^; iii) smoked; iv) evidence of infectious diseases; v) prior history of cancer and/or other chronic diseases; vi) treatment with anti-inflammatory drugs; vii) pregnant or breast-feeding females; viii) diagnosed with type 1 diabetes; ix) active liver diseases and/or significant liver dysfunction; x) renal disease; xi) autoimmune disorder; or xii) experience of severe complications, including diabetic ketoacidosis and hyperglycemic hyperosmolar status.

### Biochemical measurements

Whole blood samples were collected in K3 EDTA Vacutainer tubes after ≥8 h fasting. The samples were centrifuged for 5 min at 400 × g at room temperature (20 ± 2°C) and the plasma was collected for further assessment of biochemical parameters, including fasting blood glucose (FBG), fasting plasma insulin (FIN), total cholesterol (TC), triglycerides (TG), high-density lipoprotein cholesterol (HDL-C) and low-density lipoprotein cholesterol (LDL-C). The concentration of free fatty acids (FFAs) was determined by improved copper reagent colorimetry (Applygen Technologies Inc., Beijing, China), according to the manufacturer’s instructions. The insulin resistance (IR) was evaluated with the homeostasis model assessment (HOMA) as follows: HOMA-IR = FIN (μU/ml) × FBG (mM)/22.5 (27).

### Collection of PBMCs

PBMCs were isolated from the heparinized peripheral blood of 64 participants over Ficoll-Hypaque density gradient centrifugation (Pharmacia Biotech, Piscataway, NJ, USA), following the manufacturer’s instructions. The resultant PBMCs were used for quantitative polymerase chain reaction (qPCR) analysis.

### Culture and treatment of PBMCs

PBMCs, isolated from the healthy subjects, were cultivated in RPMI 1640 complete culture medium (Gibco Life Technologies, Grand Island, NY, USA), supplemented with 10% fetal bovine serum (Gibco Life Technologies), 1% penicillin-streptomycin (Gibco Life Technologies) and 5.6 mM glucose at 37.0°C in a humidified atmosphere (5% CO_2_, 95% air). Following overnight culture in six-well plates, the PBMCs were incubated with and without 250 μM PA (Sigma-Aldrich, St. Louis, MO, USA) for 2 h. The cells were harvested for RNA and protein expression analysis, and the supernatant was collected for TNF-α and IL-6 measurement by ELISA.

### RNA isolation and reverse transcription qPCR

Total RNA from the PBMCs was extracted with TRIzol reagent (Takara Bio, Inc., Shiga, Japan) and cDNA was generated by Moloney Murine Leukemia Virus reverse transcriptase (Promega Corporation, Madison, WI, USA), according to the manufacturer’s instructions. qPCR was performed using a SYBR Green PCR mix kit (Takara Bio, Inc.), following the manufacturer’s instructions. The primers used were as follows: NR4A1, 5′-CCAGCACTGCCAAACTGGACTA-3′ (forward) and 5′-CTCAGCAAAGCCAGGGATCTTC-3′ (reverse) (28); and β-actin, TCTACAATGAGCTGCGTGTG (forward) and GGTGAGGATCTTCATGAGGT (reverse). Following an initial denaturation step at 95°C for 30 sec, 40 PCR cycles consisting of 5 sec at 95°C, 30 sec at 58°C and 30 sec at 72°C were conducted. The qPCR data were normalized against the levels of β-actin mRNA and analyzed using ABI StepOne™ Data Analysis software (Applied Biosystems, Foster City, CA, USA).

### Analysis of NR4A1 protein using Western blotting

Total protein from the PBMCs and cultured PBMCs of the participants was extracted with radioimmunoprecipitation assay buffer (Beyotime Institute of Biotechnology, Haimen, China), supplemented with 1% phosphatase and protease inhibitor cocktails (Thermo Fisher Scientific, Waltham, MA, USA). The protein concentration was measured using a bicinchoninic acid kit (Thermo Fisher Scientific), according to the manufacturer’s instructions. The lysates with equal amounts of protein were electrophoresed by SDS-PAGE, and subsequent transblotting and immunodetection were conducted, as described previously (29). Primary antibodies against NR4A1 (Bioworld Technology, Inc., St. Louis Park, MN, USA) and Glyceraldehyde 3-phosphate dehydrogenase (GAPDH; Santa Cruz Biotechnology, Inc., Santa Cruz, CA, USA) were used. The intensity of the bands was determined by Image J 2× software (National Institutes of Health, Bethesda, MD, USA) and normalized with GAPDH.

### Measurement of inflammatory cytokines

The concentrations of TNF-α and IL-6 in the plasma or cell supernatant were measured with human TNF-α and IL-6 ELISA kits (Boster Biological Technology, Ltd., Wuhan, China.), according to the manufacturer’s instructions.

### Statistical analysis

Statistical analysis was performed using SPSS 20 software (IBM, Armonk, NY, USA), and the data are expressed as the mean ± standard deviation. The χ^2^ test and Student’s t-test of independent samples were used to compare the differences between two groups. Pearson’s correlation analysis was used to identify linear correlations between variables. P<0.05 was considered to indicate a statistically significant difference (^*^P<0.05 and ^**^P<0.01). The graphs were performed using Graphpad prism 6.0 (GraphPad Software, San Diego, CA, USA) and Sigmaplot (Systat Software, Inc. San Jose, CA, USA) (^*^P<0.05 and ^**^P<0.01).

## Results

### Clinical parameters of the subjects

**Figure 1:**
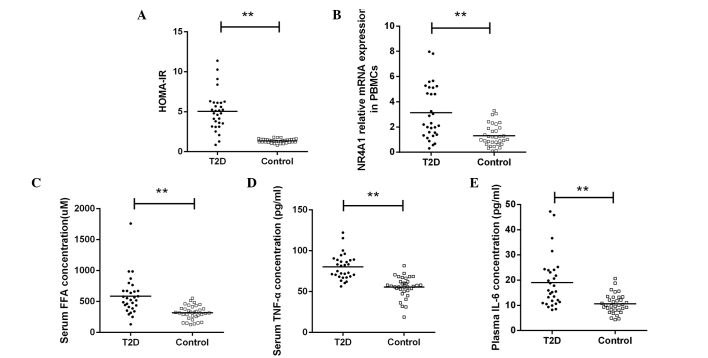
Comparison of various parameters between patients with T2D and healthy controls. (A) HOMA-IR; (B) NR4A1 mRNA relative expression, normalized against β-actin; (C) serum concentration of FFAs; (D) plasma concentration of TNF-α; and (E) plasma concentration of IL-6. ^*^P<0.05 and ^**^P<0.01. NR4A1, nuclear receptor subfamily 4 group A member 1; HOMA-IR, homeostasis model assessment of insulin resistance; FFAs, free fatty acids; TNF-α, tumor necrosis factor-α; IL-6, interleukin-6; T2D, type 2 diabetes; PBMCs, peripheral blood mononuclear cells.

A summary of the clinical parameters of the subjects enrolled in the study is shown in Table I. No statistically significant differences were observed between the T2D and control groups with regard to age, gender, blood pressure or liver or kidney function. As expected, statistically significant differences were identified in the BMI, FBG, FIN, TG, TC, LDL-C and HDL-C in patients with T2D compared with the control group. A statistically significant increase was also observed in the HOMA-IR for T2D patients (5.06±2.41 vs. 1.35±0.25, P<0.01; Fig. 1A). Although an increase in the leukocyte count was observed in T2D patients in previous trials, as aforementioned (10,11), in the current study, the peripheral total leukocyte and differential counts did not exhibit a statistically significant difference between the two groups.

### Transcriptional increase in NR4A1 expression in PBMCs from patients with T2D

Compared with the controls, the relative mRNA expression levels of NR4A1 in the PBMCs from the patients with T2D increased (3.13±2.14 vs. 1.30±0.85, P<0.01; Fig. 1B).

### Levels of FFAs, TNF-α and IL-6 increase in the plasma of T2D patients

As expected, statistically significant differences were observed in the levels of FFAs when comparing the patients with T2D with the control group (586.58±301.93 vs. 319.07±113.41 μM, P<0.01; Fig. 1C). In addition, the plasma concentrations of TNF-α (80.12±15.51 vs. 53.62±11.14 pg/ml, P<0.01; Fig. 1D) and IL-6 (19.08±10.19 vs*.* 9.82±3.05 pg/ml, P<0.01; Fig. 1E) were increased in the patients with T2D, as compared with the control subjects.

### TNF-α and IL-6 levels increase in the supernatant of PA-stimulated PBMCs from healthy participants

**Figure 2:**
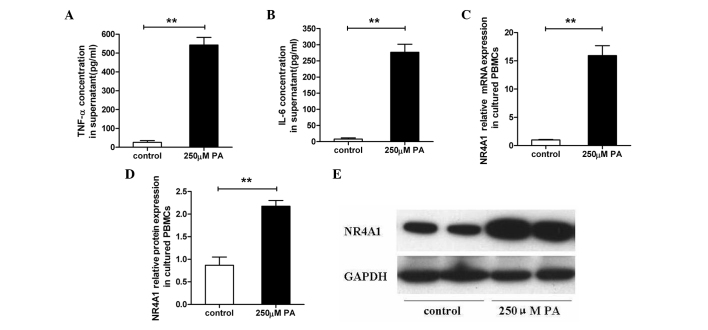
Cultured PBMCs from healthy controls were treated with or without 250 μM PA. (A) Concentration of (A) TNF-α and (B) IL-6 in the supernatant of the PBMCs (pg/ml). (C) Quantitative reverse transcription polymerase chain reaction was performed to detect the relative mRNA expression levels of NR4A1 after 2 h of stimulation with 250 μM PA, normalized against β-actin. Results of the Western blot analysis. (D) Graph and (E) immunoblot showing NR4A1 protein expression in the PBMCs. ^*^P<0.05 and ^**^P<0.01. NR4A1, nuclear receptor subfamily 4 group A member 1; PMBCs, peripheral blood mononuclear cells; TNF-α, tumor necrosis factor-α; IL-6, interleukin-6; PA, palmitic acid.

In the PBMCs from the healthy subjects, the supernatants were collected following treatment with or without 250 μM PA for 2 h. Subsequently, the protein concentrations of TNF-α (543.31±40.08 vs*.* 26.09±9.35 pg/ml, P<0.01; Fig. 2A) and IL-6 (276.82±24.88 vs*.* 7.80±3.45 pg/ml, P<0.01; Fig. 2B) were analyzed and were found to be significantly induced by 250 μM PA stimulation.

### PA induces NR4A1 expression in cultured PBMCs from healthy participants

In the cultured PBMCs from the healthy subjects, NR4A1 mRNA relative expression increased in the 250 μM PA-induced PBMCs when compared with the controls (15.92±1.75 vs. 1.00±0.09, P<0.01, Fig. 2C), as well as the NR4A1 relative protein expression level (2.18±0.12 vs. 0.87±0.18, P<0.01, Fig. 2D and E), as determined by qPCR and western blot analysis.

### Correlation between NR4A1 mRNA expression and other parameters

**Figure 3:**
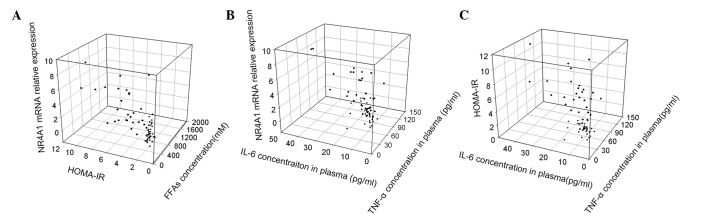
Correlation analyses among (A) the NR4A1 mRNA expression level, HOMA-IR and FFAs concentration; (B) the NR4A1 mRNA expression level and TNF-α and IL-6 concentrations; and (C) HOMA-IR and the TNF-α and IL-6 concentrations. NR4A1, nuclear receptor subfamily 4 group A member 1; HOMA-IR, homeostasis model assessment of insulin resistance; FFAs, free fatty acids; TNF-α, tumor necrosis factor-α; IL-6, interleukin-6.

Using Pearson’s correlation analysis, several positive correlations were identified among the parameters. The NR4A1 mRNA relative expression level was found to exhibit a positive correlation with several diabetes-related parameters, including the HOMA-IR (r=0.761, P<0.01; Fig. 3A), FFAs (r=0.560, P<0.01; Fig. 3A), TNF-α (r=0.697, P<0.01; Fig. 3B) and IL-6 levels (r=0.796 P<0.01, Fig. 3B). The HOMA-IR was also shown to positively correlate with the level of FFAs (r=0.513, P<0.01; Fig. 3A), TNF-α (r=0.728, P<0.01; Fig. 3C) and IL-6 (r=0.590, P<0.01; Fig. 3C). In addition, statistically significant correlations were observed between the levels of FFAs and the protein expression levels of TNF-α (r=0.475, P<0.001) and IL-6 (r=0.402, P=0.001). However, there were no evident correlations between the total leukocyte/differential count and other variables, including NR4A1 mRNA expression, HOMA-IR, FFAs, TNF-α and IL-6.

## Discussion

T2D is widely recognized as not only a metabolic disorder, but is also characterized by a chronic inflammatory state. Similar to previous studies reporting alterations in inflammatory biomarkers in patients diagnosed with T2D (3–7,10,11), similar findings were obtained in the present study on PBMCs. The concentration of TNF-α and IL-6 in the plasma was significantly higher in patients with T2D compared with the healthy controls, which further indicated the association between T2D and inflammation. However, no statistically significant difference was identified in the total leukocyte or differential count between patients with T2D and healthy subjects in the current study.

NR4A1, a member of the NR4A orphan nuclear receptor family, has received increasing attention due to its effects on metabolic regulation, with outcomes affecting glucose metabolism, lipolysis and energy expenditure (30–32). The insulin response of the liver and skeletal muscle was demonstrated to be blunted in NR4A1-deficient mice fed with a high-fat diet (31). In the liver, NR4A1 induced the expression of gluconeogenesis-related genes and increased glucose production (32). In addition, other studies have shown that NR4A1 is involved in the impairment of islet β-cells. A study by Briand *et al* (33) on murine pancreatic β-cells demonstrated that fatty acids and cytokine treatment induced NR4A1 expression, and NR4A1 overexpression decreased insulin secretion. Therefore, NRF4A1 exhibits effects on insulin function and insulin secretion, which are commonly recognized as two major pathophysiological bases of T2D.

NR4A1 has been reported to be involved in the inflammatory disease, atherosclerosis. For instance, higher expression levels of NR4A1 were detected in macrophages in human atherosclerotic lesions (24,25). Similarly, the present study demonstrated that NR4A1 mRNA expression is increased in the PBMCs of patients with T2D, as compared with the healthy participants. Furthermore, mRNA expression levels of NR4A1 were shown to positively correlate with the levels of FFAs, TNF-α and IL-6, as well as the HOMA-IR. *In vitro*, the expression of NR4A1 is highly inducible in macrophages using diverse inflammatory stimuli, including oxLDL, LPS and TNF-α (23,24,26). You *et al* (22) found that NR4A1 protein expression was upregulated in human ECs by TNF-α in a time- and concentration-dependent manner. In accordance with these studies, the present study demonstrated that NR4A1 expression in cultured PBMCs was strongly upregulated by 250 μM PA stimulation, as determined by qPCR and western blot analysis.

Therefore, NR4A1 may be involved in the inflammatory process and may be associated with inflammatory disease. With regard to the potential mechanism of NR4A1 induction, certain studies have provided insights. Shao *et al* (34) reported that the mitogen-activated protein kinase (MAPK) signaling pathway mediated the induction of NR4A1 in Raw264.7 cells in response to an oxLDL stimulus. Treatment with a p38 MAPK-specific inhibitor on oxLDL-induced Raw264.7 cells was shown to attenuate NR4A1 expression. Further study indicated that NR4A1 suppressed macrophages to uptake oxLDL and inhibit proinflammatory activation, which subsequently decreased macrophage activation.

The aforementioned studies (23,24,26) demonstrated that NR4A1 was induced by multiple inflammatory cytokines and processed the function to suppress inflammation. However, the potential mechanism through which NR4A1 inhibits inflammatory activity is not completely clear. In a further study (22) that investigated the detailed mechanisms underlying the suppression of inflammatory activity by NR4A1, a role in the nuclear factor (NF)-κB pathway was revealed. The NF-κB signaling pathway is known to play an important role in inflammation. In stimulated cells, IKK degrades IκB molecules, including IκBα, which inhibits the NF-κB pathway by forming inactivated complexes with NF-κB. NF-κB is subsequently released from the cytoplasm to the nucleus, where the molecule activates target genes (35).

An *in vitro* experiment by Hong *et al* (36) suggested that NR4A1 directly interacts with the p65 subunit of NF-κB through its C-terminal region. Pei *et al* (23,26) also identified NR4A1 as an NF-κB-responsive gene in macrophages. Furthermore, You *et al* (22) found that adenovirus-mediated overexpression of NR4A1 markedly attenuated basal, TNF-α- and IL-1β-stimulated NF-κB promoter activity in a dose-dependent manner. NR4A1 also exhibited dose-dependent upregulation of IκBα expression, which subsequently inhibited the translocation of NF-κB.

In early studies, NR4A1 was shown to regulate gene transcription by binding to the NGFI-B response element (NBRE; AAAGGTCA) (37), which has been shown to exist in the human IκBα promoter. Mutation of the NBRE site eliminated the responsiveness of the human IκBα promoter to NR4A1, indicating that this site mediates IκBα transcriptional induction by NR4A1 (22). The induction of NR4A1 by proinflammatory signals was hypothesized to generate an additional negative feedback loop in the NF-κB signaling pathway (38).

Therefore, the orphan nuclear receptor, NR4A1, is induced in response to inflammatory stimuli, including TNF-α, LPS, oxLDL and FFAs, and may be induced via the p38 MAPK signaling pathway. The receptor subsequently exerts an anti-inflammatory function by suppressing NF-κB activity via the induction of IκBα expression. NR4A1 functions as a negative regulator by inhibiting NF-κB activation. The regulation, expression and activity of NR4A1 are hypothesized to represent a potential target for the prevention and treatment of inflammatory diseases.

In conclusion, the present study demonstrated that the expression of NR4A1 is increased in PBMCs from patients that have been newly diagnosed with T2D. In addition, NR4A1 expression was shown to significantly correlate with the levels of the inflammatory cytokines, TNF-α and IL-6, as well as the diabetes-related parameters, FIN, FBG, HOMA-IR and FFA. Therefore, NR4A1 is associated with the inflammatory state in T2D. However, understanding the specific role of NR4A1 in the regulation of inflammation in diabetes requires further study.

## Figures and Tables

**Table I:** Clinical parameters of the subjects.

Variables	Type 2 diabetes group	Control group	P-value
Age (years)	49.00±7.68	44.94±10.94	0.088
Gender, male/female (n)	21/13	17/13	0.681
BMI (kg/m^2^)	26.20±2.67	23.66±3.11	<0.01
Systolic pressure (mmHg)	128.60±11.08	122.94±12.17	0.057
Alanine aminotransferase (U/l)	33.47±15.06	28.06±11.26	0.106
Blood urea nitrogen (mM)	5.30±1.45	4.81±1.17	0.141
Serum creatinine (μM)	71.38±13.73	77.74±15.11	0.084
FBG (mM)	8.50±2.50	5.26±0.29	<0.01
FIN (μU/ml)	13.69±5.73	5.81±1.05	<0.01
TG (mM)	1.91±0.81	1.44±0.97	<0.05
TC (mM)	5.41±1.08	4.81±0.81	<0.05
HDL-C (mM)	1.06±0.16	1.21±0.17	<0.01
LDL-C (mM)	3.65±0.92	3.10±0.76	<0.05
White blood cell count (×10^9^)	6.80±1.82	6.38±1.57	0.326
Neutrophil (%)	56.20±8.41	56.95±8.41	0.724
Lymphocyte (%)	33.48±8.57	32.47±8.10	0.630
Monocyte (%)	7.64±1.87	7.25±1.50	0.358

BMI, body mass index; FBG, fasting blood glucose; FIN, fasting plasma insulin; TG, triglyceride; TC, total cholesterol; HDL-C, high-density lipoprotein-cholesterol; LDL-C, low-density lipoprotein-cholesterol.
